# Impact of the prognostic nutritional index on the recompensation of patients with decompensated primary biliary cholangitis

**DOI:** 10.3389/fimmu.2025.1605380

**Published:** 2025-10-01

**Authors:** Chunjiao Yu, Fan Ni, Mengyao Zheng, Zhaoda Duan, Wenjie Yang, Man Huang, Hongyan Xu, Mengyu Li, Shunyu Chen, Chaozhang Gu, Jinhui Yang, Jianjun Liu, Hongtao Lei, Shan Yan

**Affiliations:** ^1^ Yunnan Key Laboratory of Breast Cancer Precision Medicine and Academy of Biomedical Engineering, School of Public Health, Kunming Medical University, Kunming, Yunnan, China; ^2^ Department of Hepatopancreatobiliary (HPB), Thyroid and Vascular Surgery, Yuxi People’s Hospital, Yuxi, Yunnan, China; ^3^ Department of Gastroenterology, The Second Affiliated Hospital of Kunming Medical University, Kunming, Yunnan, China; ^4^ School of Basic Medicine, Kunming Medical University, Kunming, Yunnan, China; ^5^ Department of Radiation Oncology, The First Affiliated Hospital of Kunming Medical University, Kunming, Yunnan, China; ^6^ School of Clinical Oncology, Kunming Medical University, Kunming, Yunnan, China; ^7^ Key Laboratory of Integrated Care for Geriatric Chronic Diseases School of Nursing, Kunming Medical University, Kunming, Yunnan, China

**Keywords:** prognostic nutritional index, primary biliary cholangitis, decompensated liver cirrhosis, recompensation, association

## Abstract

**Background:**

Nutrition and immunity are associated with the progression of primary biliary cholangitis (PBC); however, the connection between these factors and the prognosis of patients with decompensated PBC remains unclear.

**Methods:**

This study adopted a retrospective cohort design, enrolling patients with decompensated PBC who received standard-dose ursodeoxycholic acid treatment. The prognostic nutritional index (PNI) was calculated based on lymphocyte count and albumin levels at admission. The Cox proportional hazards model was used to analyze the impact of PNI on the recompensation of patients with decompensated liver cirrhosis.

**Results:**

A total of 413 patients with decompensated PBC were included, with a mean age of 60.34 ± 11.57 years, of whom 343 (83.1%) were female. During the follow-up period, 119 patients (28.8%) achieved recompensation. The median baseline PNI was higher in the recompensation group [44.5, interquartile range (IQR): 39.5–49.3] than in the non-recompensation group (33.9, IQR: 29.8–38.2). In the fully adjusted model, a higher baseline PNI score was associated with an increased probability of recompensation [hazard ratio (HR): 1.11, 95% CI: 1.08–1.15]. Subgroup analysis showed that baseline PNI was significantly associated with recompensation in patients with ascites (HR: 1.15, 95% CI: 1.11–1.20), without ascites (HR: 1.06, 95% CI: 1.01–1.12), with bleeding (HR: 1.10, 95% CI: 1.03–1.17), and without bleeding (HR: 1.13, 95% CI: 1.08–1.17). Trend analysis showed that PNI, as a continuous variable, was significantly positively correlated with the likelihood of recompensation (HR: 1.11, 95% CI: 1.08–1.15). Sensitivity analysis confirmed that after excluding patients with comorbidities, the association between PNI and recompensation remained stable (HR: 1.11, 95% CI: 1.07–1.15).

**Conclusions:**

PNI is an independent protective factor for recompensation in patients with decompensated PBC.

## 1 Introduction

Primary biliary cholangitis (PBC) is a disease characterized by the destruction of intrahepatic small bile ducts and progressive cholestasis, which may eventually lead to liver cirrhosis and liver failure (1–3). Ursodeoxycholic acid (UDCA) is the first-line treatment for PBC (4) and has been proven to significantly reduce the risk of liver transplantation or death (5). As PBC progresses to its late stage, patients may develop decompensated liver cirrhosis, which significantly impacts their quality of life and survival rate (6, 7). Although patients with decompensated PBC have a poor prognosis, some can recover from a decompensated state to a compensated state through effective treatment and management, achieving recompensation (8–10). Recompensation not only improves long-term survival rates but also reduces the incidence of complications. Therefore, achieving a compensated state is a crucial treatment goal for patients with decompensated PBC (11).

Malnutrition and immune dysfunction are common in patients with decompensated liver cirrhosis, both of which play crucial roles in disease progression and prognosis (12, 13). Malnutrition exacerbates liver function impairment and increases the risk of infections and other complications (14, 15), while immune dysfunction further promotes liver disease progression and reduces patients’ response to treatment (16). Importantly, studies have confirmed that adequate nutritional support can improve survival in patients with decompensated cirrhosis. For instance, Vilar Gomez et al. (17) demonstrated that a nutritional supplement improved survival and slowed disease progression in patients with Hepatitis C Virus (HCV)-related decompensated cirrhosis. Similarly, Muto et al. (18) showed that oral branched-chain amino acid granules improved event-free survival in cirrhotic patients, highlighting the therapeutic potential of nutritional intervention. The prognostic nutritional index (PNI), calculated from serum albumin and peripheral lymphocyte count, is a simple yet effective composite marker of nutritional and immune status (19). Previous studies have linked higher PNI levels with improved survival in cirrhotic patients (20), suggesting that PNI may serve as a valuable prognostic indicator. However, despite its growing use in liver disease, the potential role of PNI in predicting recompensation in patients with decompensated PBC has not been fully elucidated. Therefore, this study aimed to evaluate the association between baseline PNI and recompensation in patients with decompensated PBC receiving UDCA therapy and to explore the clinical value of PNI in guiding risk stratification and therapeutic decisions.

## 2 Methods

### 2.1 Study design and subjects

This study was a retrospective cohort analysis that included patients diagnosed with PBC at the Second Affiliated Hospital of Kunming Medical University from January 2013 to December 2023. The study was approved by the Ethics Committee of the Second Affiliated Hospital of Kunming Medical University (YJ-2023-96).

The diagnosis of PBC was established based on the European Association for the Study of the Liver (EASL) Clinical Practice Guidelines (4) and the American Association for the Study of Liver Diseases (AASLD) Practice Guidance (2), requiring at least two of the following criteria: elevated serum alkaline phosphatase (ALP), the presence of antimitochondrial antibodies (AMA), or histological features consistent with non-suppurative destructive cholangitis and interlobular bile duct loss. Decompensated PBC was defined as PBC with one or more complications of cirrhosis, including ascites, variceal bleeding, or hepatic encephalopathy, as recommended by the EASL guidelines for decompensated cirrhosis (3). The inclusion criteria were as follows: 1) diagnosis consistent with the above PBC criteria; 2) diagnosed with decompensated PBC; 3) received effective etiological treatment, defined as standard-dose UDCA at 13–15 mg·kg^−1^·day^−1^ for at least 6 months, based on current guidelines (2, 4); and 4) had complete follow-up data. The exclusion criteria were as follows: 1) presence of malignant tumors, 2) portal vein thrombosis, 3) previous transjugular intrahepatic portosystemic shunt, 4) history of liver transplantation, 5) incomplete clinical data, and 6) presence of acute infection.

### 2.2 Data collection

1. Demographic and clinical data: Patient information, including age, gender, body mass index (BMI), and history of underlying diseases (such as hypertension, diabetes, and coronary heart disease), was collected.
2. Laboratory indicators: Serum albumin, lymphocyte count, total bilirubin, alanine aminotransferase (ALT), aspartate aminotransferase (AST), and ALP, among others, were recorded.
3. PNI calculation formula: PNI = Albumin (g/L) + 5 × Lymphocyte count (10^9^/L).

### 2.3 Outcome

Recompensation was defined as follows: when no clinical events such as ascites, variceal bleeding, or hepatic encephalopathy occurred during a continuous 12-month follow-up period, accompanied by stable liver function (21).

### 2.4 Statistical analysis

Data analysis in this study was performed using SPSS 26.0 (IBM Corp., Armonk, New York, USA). Normally distributed data are presented as mean ± SD, and comparisons between groups were performed using the independent samples t-test. Non-normally distributed data are expressed as the median and interquartile range [M (IQR)], and group differences were analyzed using the Mann–Whitney U test. Categorical variables are presented as frequency and percentage [n (%)], and comparisons between groups were performed using the chi-square test or Fisher’s exact test, as appropriate. The Cox proportional hazards regression model was used to evaluate the impact of the prognostic nutritional index on recompensation in patients with decompensated primary biliary cholangitis. Hazard ratios (HRs) and 95% confidence intervals (CIs) were used to describe the strength of the association. The proportional hazards assumption was tested using Schoenfeld residuals and log–log survival curves. Subgroup analysis was stratified based on baseline characteristics (presence or absence of ascites and gastrointestinal bleeding) to validate the robustness of the results in different populations. Sensitivity analysis was conducted by excluding patients with diabetes, hypertension, or coronary artery disease to eliminate the potential confounding effects of these chronic conditions on liver recompensation. Trend analysis: The study population was first divided into tertiles (T1, T2, and T3) based on PNI values, and the HR and 95% CI for each tertile were calculated relative to T1. Subsequently, PNI tertiles were treated as a continuous variable for trend testing, and the trend p-value was calculated to assess the dose–response relationship. All statistical tests were two-sided, and a p-value <0.05 was considered statistically significant.

## 3 Results

### 3.1 Baseline characteristics of patients

A total of 413 patients with decompensated PBC were included in this study, with a mean age of 60.34 ± 11.57 years. Among them, 343 (83.1%) were female. The median BMI was 21.2 kg/m^2^ (IQR: 19.2–23.5). Recompensation occurred in 119 (28.8%) patients. Comparison of baseline characteristics between the recompensation group and the persistent decompensation group showed that BMI, albumin, lymphocyte count, and PNI were higher in the recompensation group, whereas total bilirubin, AST, and the proportion of ascites were lower than those in the persistent decompensation group (all p < 0.05) (**Table 1**).

**Table 1:** Comparison of clinical characteristics between recompensated and persistently decompensated patients (n = 413).

Characteristic	Total (n = 413)	Recompensation (n = 119)	Non-recompensation (n = 294)	P-value
Age (years), mean ± SD	60.34 ± 11.57	58.73 ± 11.51	60.99 ± 11.55	0.070
Gender, n (%)				0.038
Male	70 (16.9)	13 (10.9)	57 (19.4)	
Female	343 (83.1)	106 (89.1)	237 (80.6)	
BMI (kg/m^2^), M (IQR)	21.2 (19.2, 23.5)	21.8 (18.6, 23.0)	20.8 (20.5, 24.2)	<0.001
Hypertension, n (%)				0.252
No	341 (82.6)	94 (79.0)	247 (84.0)	
Yes	72 (17.4)	25 (21.0)	47 (16.0)	
Coronary heart disease, n (%)				0.339
No	401 (97.1)	114 (95.8)	287 (97.6)	
Yes	12 (2.9)	5 (4.2)	7 (2.4)	
Diabetes, n (%)				0.109
No	362 (87.7)	106 (89.1)	256 (87.1)	
Yes	51 (12.3)	13 (10.9)	38 (12.9)	
Fatty liver, n (%)				0.109
No	406 (98.3)	115 (96.6)	291 (99.0)	
Yes	7 (1.7)	4 (3.4)	3 (1.0)	
Hepatic encephalopathy, n (%)				0.564
No	389 (94.2)	116 (97.5)	273 (92.9)	
Yes	24 (5.8)	3 (2.5)	21 (7.1)	
Bleeding, n (%)				0.061
No	284 (68.8)	90 (75.6)	194 (66.0)	
Yes	129 (31.2)	29 (24.4)	100 (44.0)	
Ascites, n (%)				<0.001
No	67 (16.2)	40 (33.6)	27 (9.2)	
Yes	346 (83.8)	79 (66.4)	267 (90.8)	
TBIL (μmol/L), M (IQR)	30.7 (17.2, 76.4)	17.7 (14.1, 27.9)	40.9 (22.7, 112.6)	<0.001
ALT (u/L), M (IQR)	41 (24, 74.5)	37 (22, 75)	43 (25, 74.3)	0.290
AST (u/L), M (IQR)	67 (39, 103.5)	55 (32, 81)	72.5 (42, 113.3)	0.001
ALP (u/L), M (IQR)	198 (129, 338)	171 (122, 301)	213.5 (130.8, 347.8)	0.095
Albumin (g/dL), M (IQR)	30.9 (26.7, 36.0)	37.7 (34.0, 40.8)	29.20 (24.9, 29.2)	<0.001
Absolute lymphocyte count (/mm^3^), M (IQR)	1.02 (0.69, 1.46)	1.25 (0.85, 1.80)	0.95 (0.66, 1.32)	<0.001
PNI, M (IQR)	36.2 (31.5, 42.5)	44.5 (39.5, 49.3)	33.9 (29.8, 38.2)	<0.001

BMI, body mass index; TBIL, total bilirubin; ALT, alanine aminotransferase; AST, aspartate aminotransferase; ALP, alkaline phosphatase; PNI, prognostic nutritional index.

### 3.2 PNI as a factor influencing recompensation in patients with decompensated PBC

Univariate Cox hazards analysis showed that a higher PNI was significantly associated with a greater likelihood of achieving recompensation (HR: 1.13, 95% CI: 1.10–1.15). In the multivariate Cox hazards regression analysis, Model A (HR: 1.13, 95% CI: 1.10–1.15), Model B (HR: 1.12, 95% CI: 1.09–1.15), and Model C (HR: 1.11, 95% CI: 1.08–1.15) all showed a significant association between higher PNI and a greater likelihood of achieving recompensation (**Table 2**).

**Table 2:** Factors associated with a higher probability of recompensation.

Variable	Univariate analysis		Multivariate analysis					
			Model A		Model B		Model C	
	HR (95% CI)	P-value	HR (95% CI)	P- value	HR (95% CI)	P-value	HR (95% CI)	P-value
Age (years)	0.99 (0.97, 1.00)	0.094	1.00 (0.99, 1.00)	0.600	1.00 (0.98, 1.02)	0.860	0.99 (0.98, 1.01)	0.475
Gender								
Male	Reference		Reference		Reference		Reference	
Female	1.75 (0.98, 3.11)	0.057	1.54 (0.87, 2.76)	0.142	1.55 (0.87, 2.79)	0.141	1.34 (0.73, 2.44)	0.345
BMI (kg/m^2^)	1.08 (1.03, 1.13)	0.010	1.07 (1.02, 1.13)	0.010	1.08 (1.02, 1.14)	0.008	1.05 (0.99, 1.12)	0.114
Hypertension								
No	Reference				Reference		Reference	
Yes	1.31 (0.85, 2.04)	0.238			0.82 (0.49, 1.35)	0.482	0.84 (0.51, 1.39)	0.504
Coronary heart disease								
No	Reference				Reference		Reference	
Yes	1.67 (0.68, 4.09)	0.264			2.37 (0.89, 6.32)	0.085	2.10 (0.78, 5.60)	0.142
Diabetes								
No	Reference				Reference		Reference	
Yes	0.85 (0.48, 1.51)	0.583			0.89 (0.48, 1.63)	0.694	0.87 (0.47, 1.60)	0.640
Fatty liver								
No	Reference				Reference		Reference	
Yes	2.44 (0.90, 6.61)	0.080			1.31 (0.45, 3.81	0.618	1.41 (0.48, 4.14)	0.537
Hepatic encephalopathy								
No	Reference				Reference		Reference	
Yes	0.47 (0.15, 1.49)	0.201			0.81 (0.25, 2.65)	0.726	0.77 (0.24, 2.55)	0.672
Bleeding								
No	Reference				Reference		Reference	
Yes	0.69 (0.45, 1.05)	0.080			0.66 (0.43, 1.03)	0.067	0.70 (0.45, 1.08)	0.106
Ascites								
No	Reference				Reference		Reference	
Yes	0.35 (0.24, 0.51)	<0.001			0.74 (0.48, 1.15)	0.182	0.81 (0.53, 1.25)	0.350
TBIL (μmol/L)	0.98 (0.98, 0.99)	<0.001					0.99 (0.98, 1.00)	0.006
ALT (u/L), per 100 units	1.05 (0.84, 1.31)	0.650					0.84 (0.52, 1.36)	0.479
AST (u/L), per 100 units	0.77 (0.56, 1.07)	0.123					1.22 (0.65, 2.29)	0.529
ALP (u/L), per 100 units	0.96 (0.89, 1.04)	0.290					0.95 (0.86, 1.06)	0.366
PNI	1.13 (1.10, 1.15)	<0.001	1.13 (1.10, 1.15)	<0.001	1.12 (1.09, 1.15)	<0.001	1.11 (1.08, 1.15)	<0.001

BMI, body mass index; TBIL, total bilirubin; ALT, alanine aminotransferase; AST, aspartate aminotransferase; ALP, alkaline phosphatase; PNI, prognostic nutritional index.

Model A: adjusted for age, sex, and BMI.

Model B: Model A + hypertension, diabetes, coronary heart disease, fatty liver, hepatic encephalopathy, bleeding, and ascites.

Model C: Model B + TBIL, ALT, AST, and ALP.

### 3.3 Results of subgroup and sensitivity analyses

Subgroup analysis results showed that in the full model, higher PNI was significantly associated with a greater likelihood of recompensation in both patients with ascites (HR: 1.15, 95% CI: 1.11–1.20) and those without ascites (HR: 1.06, 95% CI: 1.01–1.12) (**Table 3**). Similarly, higher PNI was significantly associated with recompensation in patients with bleeding (HR: 1.10, 95% CI: 1.03–1.17) and those without bleeding (HR: 1.13, 95% CI: 1.08–1.17) (**Table 4**).

**Table 3:** Subgroup analysis of PNI as a factor influencing recompensation in patients with decompensated PBC (ascites/no ascites).

Variable	Univariate analysis		Multivariate analysis					
			Model A		Model B		Model C	
	HR (95% CI)	P-value	HR (95% CI)	P-value	HR (95% CI)	P-value	HR (95% CI)	P-value
Ascites								
PNI	1.17 (1.13, 1.20)	<0.001	1.16 (1.13, 1.20)	<0.001	1.17 (1.13, 1.21)	<0.001	1.15 (1.11, 1.20)	<0.001
No ascites								
PNI	1.06 (1.02, 1.10)	0.003	1.06 (1.02, 1.10)	0.002	1.05 (1.01, 1.09)	0.009	1.06 (1.01, 1.12)	0.033

PNI, prognostic nutritional index; PBC, primary biliary cholangitis; BMI, body mass index; TBIL, total bilirubin; ALT, alanine aminotransferase; AST, aspartate aminotransferase; ALP, alkaline phosphatase.

Model A: adjusted for age, sex, and BMI.

Model B: Model A + hypertension, diabetes, coronary heart disease, fatty liver, hepatic encephalopathy, and bleeding.

Model C: Model B + TBIL, ALT, AST, and ALP.

**Table 4:** Subgroup analysis of PNI as a factor influencing recompensation in patients with decompensated PBC (bleeding/no bleeding).

Variable	Univariate analysis		Multivariate analysis					
			Model A		Model B		Model C	
	HR (95% CI)	P-value	HR (95% CI)	P-value	HR (95% CI)	P-value	HR (95% CI)	P-value
Bleeding								
PNI	1.10 (1.06, 1.14)	<0.001	1.11 (1.07, 1.16)	<0.001	1.10 (1.05, 1.15)	<0.001	1.10 (1.03, 1.17)	0.005
No bleeding								
PNI	1.15 (1.11, 1.18)	<0.001	1.14 (1.11, 1.18)	<0.001	1.14 (1.10, 1.18)	<0.001	1.13 (1.08, 1.17)	<0.001

PNI, prognostic nutritional index; PBC, primary biliary cholangitis; BMI, body mass index; TBIL, total bilirubin; ALT, alanine aminotransferase; AST, aspartate aminotransferase; ALP, alkaline phosphatase.

Model A: adjusted for age, sex, and BMI.

Model B: Model A + hypertension, diabetes, coronary heart disease, fatty liver, hepatic encephalopathy, and ascites.

Model C: Model B + TBIL, ALT, AST, and ALP.

Sensitivity analysis showed that in the full model, higher PNI remained significantly associated with a greater likelihood of recompensation (HR: 1.11, 95% CI: 1.07–1.15) (**Table 5**).

**Table 5:** Sensitivity analysis of PNI as a factor influencing recompensation in patients with decompensated PBC.

Variable	Univariate analysis		Multivariate analysis					
			Model A		Model B		Model C	
	HR (95% CI)	P-value	HR (95% CI)	P-value	HR (95% CI)	P-value	HR (95% CI)	P-value
Age (years)	0.98 (0.96, 1.00)	0.050	1.01 (0.99, 1.03)	0.404	1.01 (0.99, 1.03)	0.493	1.00 (0.98, 1.02)	0.868
Gender								
Male	Reference		Reference		Reference		Reference	
Female	1.81 (0.87, 3.76)	0.111	1.68 (0.80, 3.50)	0.168	1.62 (0.77, 3.38)	0.203	1.33 (0.62, 2.84)	0.463
BMI (kg/m^2^)	1.10 (1.05, 1.17)	<0.001	1.10 (1.03, 1.17	0.003	1.11 (1.04, 1.19)	0.002	1.07 (0.99, 1.15)	0.078
Fatty liver								
No	Reference				Reference		Reference	
Yes	3.00 (0.74, 12.23)	0.125			1.39 (0.31, 6.30)	0.671	1.43 (0.31, 6.56)	0.648
Hepatic encephalopathy								
No	Reference				Reference		Reference	
Yes	0.37 (0.09, 1.51)	0.165			0.55 (0.13, 2.39)	0.420	0.52 (0.12, 2.29)	0.388
Bleeding								
No	Reference				Reference		Reference	
Yes	0.74 (0.46, 1.20)	0.220			0.65 (0.39, 1.08)	0.097	0.69 (0.42, 1.15)	0.153
Ascites								
No	Reference				Reference		Reference	
Yes	0.27 (0.17, 0.42)	<0.001			0.63 (0.37, 1.05)	0.074	0.69 (0.42, 1.15)	0.153
TBIL (μmol/L)	0.98 (0.97, 0.99)	<0.001					0.99 (0.98, 1.00)	0.015
ALT (u/L), per 100 units	1.06 (0.83, 1.36)	0.644					0.80 (0.48, 1.36)	0.415
AST (u/L), per 100 units	0.77 (0.53, 1.14)	0.188					1.33 (0.65, 2.72)	0.442
ALP (u/L), per 100 units	0.94 (0.85, 1.03)	0.190					0.92 (0.81, 1.05)	0.214
PNI	1.12 (1.10, 1.15)	<0.001	1.13 (1.10, 1.16)	<0.001	1.11 (1.08, 1.15)	<0.001	1.11 (1.07, 1.15)	<0.001

BMI, body mass index; TBIL, total bilirubin; ALT, alanine aminotransferase; AST, aspartate aminotransferase; ALP, alkaline phosphatase; PNI, prognostic nutritional index; PBC, primary biliary cholangitis.

Model A: adjusted for age, sex, and BMI.

Model B: Model A + fatty liver, hepatic encephalopathy, bleeding, and ascites.

Model C: Model B + TBIL, ALT, AST, and ALP.

### 3.4 Trend analysis

Compared to that in the T1 group, the probability of recompensation in the full model was higher in the T2 group (HR: 4.26, 95% CI: 1.63–11.13) and the T3 group (HR: 11.52, 95% CI: 4.51–29.42). Moreover, PNI, as a continuous variable, remained significantly positively correlated with the likelihood of recompensation (HR: 1.11, 95% CI: 1.08–1.15) (**Table 6**).

**Table 6:** Relationship between PNI and recompensation.

Variable	Univariate analysis		Multivariate analysis					
			Model A		Model B		Model C	
	HR (95% CI)	P-value	HR (95% CI)	P-value	HR (95% CI)	P-value	HR (95% CI)	P-value
PNI3								
T1	Reference		Reference		Reference		Reference	
T2	5.29 (2.05, 13.68)	0.001	5.50 (2.13, 14.21)	<0.001	4.88 (1.87, 12.70)	0.001	4.26 (1.63, 11.13)	0.003
T3	19.09 (7.75, 47.06)	<0.001	18.76 (7.60, 46.30)	<0.001	14.96 (5.95, 37.63)	<0.001	11.52 (4.51, 29.42)	<0.001
PNI	1.13 (1.10, 1.15)	<0.001	1.13 (1.10, 1.15)	<0.001	1.12 (1.09, 1.15)	<0.001	1.11 (1.08, 1.15)	<0.001

PNI, prognostic nutritional index; BMI, body mass index; ALT, alanine aminotransferase; AST, aspartate aminotransferase; ALP, alkaline phosphatase.

Model A: adjusted for age, sex, and BMI.

Model B: Model A + hypertension, diabetes, coronary heart disease, fatty liver, hepatic encephalopathy, bleeding, and ascites.

Model C: Model B + total bilirubin, ALT, AST, and ALP.

## 4 Discussion

This study employed a retrospective cohort analysis, enrolling 413 patients with decompensated PBC to evaluate the impact of baseline PNI on recompensation. The study found that 119 patients (%) achieved recompensation. The PNI level in the recompensation group was significantly higher than that in the persistent decompensation group. Moreover, PNI was identified as an independent protective factor for recompensation in the multivariate Cox regression analysis. Trend analysis and subgroup analysis further confirmed the robustness of these results.

The results of this study indicate that patients with higher PNI had a greater likelihood of recompensation (HR: 1.11, 95% CI: 1.08–1.15). This may be attributed to the fact that a good nutritional status and immune function support hepatocyte repair and regeneration. PNI is composed of serum albumin and lymphocyte count. Albumin reflects the patient’s nutritional status and liver synthetic function (22, 23), while lymphocytes serve as an important indicator of immune function (24–26). Consistent with the findings of Vilar et al. (17), which demonstrated that proper nutritional intervention can significantly improve the survival rate of patients with decompensated liver cirrhosis, this study also confirmed the role of nutrition in promoting recompensation. Additionally, the study by Villanueva et al. (12) demonstrated that cirrhosis-associated immune dysfunction and infection risk are key drivers of decompensation, further supporting the clinical value of PNI as a comprehensive indicator of nutritional and immune status in patients with decompensated PBC. Additionally, PNI can be used for dynamic monitoring of a patient’s recovery. Previous studies have shown that improvements in PNI are associated with increased survival rates in patients with liver cirrhosis (20). Therefore, regular monitoring of PNI and adjusting nutritional intervention strategies during treatment may help improve the rate of recompensation.

Several interrelated physiological mechanisms may explain the association between higher PNI and a greater likelihood of recompensation. Good nutritional status and immune function are essential for maintaining liver stability and supporting regeneration. Albumin reflects liver synthetic function and also has anti-inflammatory, antioxidant, and oncotic effects that help stabilize blood vessels and reduce portal hypertension. Low albumin levels have been linked to intestinal barrier dysfunction and systemic inflammation. Lymphocytes, especially CD4+ and CD8+ T cells, are crucial for immune defense and liver cell regeneration. In decompensated cirrhosis, immune dysfunction increases infection risk and worsens the disease. Therefore, a higher PNI likely indicates stronger metabolic and immune reserves, providing a better environment for liver recovery. These mechanisms highlight the clinical value of PNI not only as a prognostic marker but also as a potential target for nutritional and immune-based interventions.

The study included a large sample size, which improves the robustness of the results. We also adjusted for many confounding variables using multivariable Cox regression to strengthen the validity of the findings. However, some limitations must be acknowledged. First, as a retrospective cohort study, it is prone to selection and information bias. For example, patients with complete records and longer follow-up may have been more likely to be included, which could lead to overrepresentation of stable or compliant individuals. To reduce bias, we used predefined inclusion/exclusion criteria and a standardized data extraction process. Nonetheless, these biases may still limit the generalizability of our findings. Second, although we adjusted for many known confounders, some unmeasured factors may still affect the results. For example, Model for End-Stage Liver Disease (MELD) scores and histological staging were not consistently available. Also, we did not systematically record the use of second-line therapies. All included patients were classified as Child–Pugh class C, and due to missing International Normalized Ratio (INR) and creatinine data, MELD score calculation was not feasible. Future studies should include these variables to allow more detailed prognostic analysis. Third, we did not assess other important conditions, such as sarcopenia, frailty, or inflammation, which could influence both PNI levels and patient outcomes. These factors should be addressed in future research. Additionally, we only analyzed baseline PNI and did not evaluate how PNI changed during follow-up. Tracking PNI over time could provide more insights into nutritional and immune changes and their link to recompensation. However, inconsistent follow-up testing in this retrospective study limited our ability to conduct time-dependent analyses. Future prospective studies with regular follow-up data collection are needed to explore whether improvements in PNI can predict better outcomes.

Despite these limitations, our findings highlight the potential utility of baseline PNI as a simple yet effective marker for risk stratification and clinical decision-making in patients with decompensated PBC. Further validation in prospective multicenter cohorts and integration of time-varying PNI trends will help strengthen the clinical applicability of this index. In conclusion, PNI is an independent protective factor for recompensation in patients with decompensated PBC. Higher PNI levels are associated with better outcomes. PNI may help identify high-risk patients and guide early nutritional intervention.

## Data Availability

The original contributions presented in the study are included in the article/supplementary material. Further inquiries can be directed to the corresponding authors.

## References

[1] Tsochatzis EA Bosch J Burroughs AK. Liver cirrhosis. Lancet (London England). (2014) 383:1749–61. doi: 10.1016/S0140-6736(14)60121-5, PMID: 24480518

[2] Lindor KD Bowlus CL Boyer J Levy C Mayo M. Primary biliary cholangitis: 2018 practice guidance from the american association for the study of liver diseases. Hepatol (Baltimore Md). (2019) 69:394–419. doi: 10.1002/hep.30145, PMID: 30070375

[3] European Association for the Study of the Liver. EASL Clinical Practice Guidelines for the management of patients with decompensated cirrhosis. J Hepatol. (2018) 69:406–60. doi: 10.1016/j.jhep.2018.03.024, PMID: 29653741

[4] European Association for the Study of the Liver. EASL Clinical Practice Guidelines: The diagnosis and management of patients with primary biliary cholangitis. J Hepatol. (2017) 67:145–72. doi: 10.1016/j.jhep.2017.03.022, PMID: 28427765

[5] Chan CW Gunsar F Feudjo M Rigamonti C Vlachogiannakos J Carpenter JR. Long-term ursodeoxycholic acid therapy for primary biliary cirrhosis: a follow-up to 12 years. Alimentary Pharmacol Ther. (2005) 21:217–26. doi: 10.1111/j.1365-2036.2005.02318.x, PMID: 15691295

[6] GBD 2017 Cirrhosis Collaborators. The global, regional, and national burden of cirrhosis by cause in 195 countries and territories, 1990-2017: a systematic analysis for the Global Burden of Disease Study 2017. Lancet Gastroenterol Hepatol. (2020) 5:245–66. doi: 10.1016/S2468-1253(19)30349-8, PMID: 31981519 PMC7026710

[7] Leuschner U. Primary biliary cirrhosis–presentation and diagnosis. Clinics liver Dis. (2003) 7:741–58. doi: 10.1016/S1089-3261(03)00101-6, PMID: 14594129

[8] Poupon RE Lindor KD Cauch-Dudek K Dickson ER Poupon R Heathcote EJ. Combined analysis of randomized controlled trials of ursodeoxycholic acid in primary biliary cirrhosis. Gastroenterology. (1997) 113:884–90. doi: 10.1016/S0016-5085(97)70183-5, PMID: 9287980

[9] Heathcote EJ Cauch-Dudek K Walker V Bailey RJ Blendis LM Ghent CN. The Canadian Multicenter Double-blind Randomized Controlled Trial of ursodeoxycholic acid in primary biliary cirrhosis. Hepatol (Baltimore Md). (1994) 19:1149–56. doi: 10.1002/hep.1840190512, PMID: 8175136

[10] Xu XY Ding HG Li WG Xu JH Han Y Jia JD. Chinese guidelines on the management of liver cirrhosis (abbreviated version). World J Gastroenterol. (2020) 26:7088–103. doi: 10.3748/wjg.v26.i45.7088, PMID: 33362370 PMC7723671

[11] Hofer BS Simbrunner B Hartl L Jachs M Balcar L Paternostro R. Hepatic recompensation according to Baveno VII criteria is linked to a significant survival benefit in decompensated alcohol-related cirrhosis. Liver Int. (2023) 43:2220–31. doi: 10.1111/liv.15676, PMID: 37469291

[12] Villanueva C Albillos A Genescà J Garcia-Pagan JC Brujats A Calleja JL. Bacterial infections adversely influence the risk of decompensation and survival in compensated cirrhosis. J Hepatol. (2021) 75:589–99. doi: 10.1016/j.jhep.2021.04.022, PMID: 33905794

[13] Crisan D Procopet B Epure A Stefanescu H Suciu A Fodor A. Malnutrition and non-compliance to nutritional recommendations in patients with cirrhosis are associated with a lower survival. World J Hepatol. (2020) 12:829–40. doi: 10.4254/wjh.v12.i10.829, PMID: 33200020 PMC7643216

[14] Pinheiro J Jameel I Palejwala A. Deranged liver function tests and liver insults in malnourished patients: A report of two cases and literature review. Cureus. (2021) 13:e19607. doi: 10.7759/cureus.19607, PMID: 34956745 PMC8674153

[15] Pinedo S Solano D de la Villa FM Bilbao Goitia P. Protein-calorie malnutrition and degree of hepatic failure in chronic hepatopathy. Rev espanola enfermedades digestivas. (1993) 84:381–5., PMID: 8129992

[16] Yu R Huang Y Hu X Chen J. Analysis of machine learning based integration to identify the crosslink between inflammation and immune response in non-alcoholic fatty liver disease through bioinformatic analysis. Heliyon. (2024) 10:e32783. doi: 10.1016/j.heliyon.2024.e32783, PMID: 39108890 PMC11301246

[17] Vilar Gomez E Sanchez Rodriguez Y Torres Gonzalez A Calzadilla Bertot L Arus Soler E Martinez Perez Y. Viusid, a nutritional supplement, increases survival and reduces disease progression in HCV-related decompensated cirrhosis: a randomised and controlled trial. BMJ Open. (2011) 1:e000140. doi: 10.1136/bmjopen-2011-000140, PMID: 22021873 PMC3191588

[18] Muto Y Sato S Watanabe A Moriwaki H Suzuki K Kato A. Effects of oral branched-chain amino acid granules on event-free survival in patients with liver cirrhosis. Clin Gastroenterol Hepatol. (2005) 3:705–13. doi: 10.1016/S1542-3565(05)00017-0, PMID: 16206505

[19] Onodera T Goseki N Kosaki G. Prognostic nutritional index in gastrointestinal surgery of malnourished cancer patients. Nihon Geka Gakkai zasshi. (1984) 85:1001–5. doi: 10.1016/S2468-1253(19)30349-8, PMID: 6438478

[20] Xie Y He C Wang W. Prognostic nutritional index: A potential biomarker for predicting the prognosis of decompensated liver cirrhosis. Front Nutr. (2022) 9:1092059. doi: 10.3389/fnut.2022.1092059, PMID: 36687701 PMC9852856

[21] de Franchis R Bosch J Garcia-Tsao G Reiberger T Ripoll C. Baveno VII - Renewing consensus in portal hypertension. J Hepatol. (2022) 76:959–74. doi: 10.1016/j.jhep.2021.12.022, PMID: 35120736 PMC11090185

[22] Yan X Zhang S Jia J Yang J Song Y Duan H. Total parenteral nutrition treatment improves the nutrition status of gynecological cancer patients by improving serum albumin level. Front Med. (2021) 8:759387. doi: 10.3389/fmed.2021.759387, PMID: 35127741 PMC8810638

[23] Agrawal S Dhiman RK Limdi JK. Evaluation of abnormal liver function tests. Postgraduate Med J. (2016) 92:223–34. doi: 10.1136/postgradmedj-2015-133715, PMID: 26842972

[24] Tang G Yuan X Luo Y Lin Q Chen Z Xing X. Establishing immune scoring model based on combination of the number, function, and phenotype of lymphocytes. Aging. (2020) 12:9328–43. doi: 10.18632/aging.103208, PMID: 32396527 PMC7288950

[25] Caraceni P Tufoni M Zaccherini G Riggio O Angeli P Alessandria C. On-treatment serum albumin level can guide long-term treatment in patients with cirrhosis and uncomplicated ascites. J Hepatol. (2021) 74:340–9. doi: 10.1016/j.jhep.2020.08.021, PMID: 32853747

[26] Arvaniti V D’Amico G Fede G Manousou P Tsochatzis E Pleguezuelo M. Infections in patients with cirrhosis increase mortality four-fold and should be used in determining prognosis. Gastroenterology. (2010) 139:1246–1256, 1256.e1241-1245. doi: 10.1053/j.gastro.2010.06.019, PMID: 20558165

